# milR20 negatively regulates the development of fruit bodies in *Pleurotus cornucopiae*

**DOI:** 10.3389/fmicb.2023.1177820

**Published:** 2023-05-04

**Authors:** Yuhui Qi, Chenyang Huang, Mengran Zhao, Xiangli Wu, Guangyu Li, Yingjie Zhang, Lijiao Zhang

**Affiliations:** ^1^Institute of Agricultural Resources and Regional Planning, Chinese Academy of Agricultural Sciences, Beijing, China; ^2^Key Laboratory of Microbial Resources, Ministry of Agriculture and Rural Affairs, Beijing, China; ^3^State Key Laboratory of Efficient Utilization of Arid and Semi-arid Arable Land in Northern China, Beijing, China; ^4^College of Life Sciences, Shanxi Normal University, Taiyuan, China

**Keywords:** milR20, fruit body development, *Pleurotus cornucopiae*, comparative transcriptome, MAPK signaling pathway

## Abstract

The mechanism underlying the development of fruit bodies in edible mushroom is a widely studied topic. In this study, the role of milRNAs in the development of fruit bodies of *Pleurotus cornucopiae* was studied by comparative analyses of the mRNAs and milRNAs at different stages of development. The genes that play a crucial role in the expression and function of milRNAs were identified and subsequently expressed and silenced at different stages of development. The total number of differentially expressed genes (DEGs) and differentially expressed milRNAs (DEMs) at different stages of development was determined to be 7,934 and 20, respectively. Comparison of the DEGs and DEMs across the different development stages revealed that DEMs and its target DEGs involved in the mitogen-activated protein kinase (MAPK) signaling pathway, protein processing in endoplasmic reticulum, endocytosis, aminoacyl-tRNA biosynthesis, RNA transport, and other metabolism pathways, which may play important roles in the development of the fruit bodies of *P*. *cornucopiae*. The function of milR20, which targeted pheromone A receptor *g8971* and was involved in the MAPK signaling pathway, was further verified by overexpression and silencing in *P*. *cornucopiae*. The results demonstrated that the overexpression of milR20 reduced the growth rate of mycelia and prolonged the development of the fruit bodies, while milR20 silencing had an opposite effect. These findings indicated that milR20 plays a negative role in the development of *P*. *cornucopiae*. This study provides novel insights into the molecular mechanism underlying the development of fruit bodies in *P*. *cornucopiae*.

## 1. Introduction

MicroRNAs (miRNAs) are small non-coding RNA molecules that are 18–24 nt long, play important regulatory roles in gene regulation, and influence various biological processes in plants and animals. Numerous miRNAs of plants and animals have been identified to date. The first miRNA-like fungal RNAs (milRNAs) were discovered in *Neurospora crassa* (Lee et al., 2010) and the milRNAs have been subsequently identified in other filamentous fungi and basidiomycetes (Kang et al., 2013; Wang et al., 2021). The characteristics of fungal milRNAs are similar to those of plant and animal miRNAs. For instance, the miRNA precursors of plants and animals have a typical hairpin structure that is similar to those of fungi. However, the biosynthesis mechanism of fungal miRNAs are different from the animal and plant miRNAs. The milRNAs of *N*. *crassa* are produced by four different mechanisms that include a distinct combination of factors, including Dicers, Argonaute protein QDE-2, the exonuclease QIP, and the RNAse III domain-containing MRPL3 protein. While the miRNA in animals and plants were produced by Dicer-like enzymes or Drosha proteins in miRNA maturation (Jones-Rhoades et al., 2006; Lee et al., 2010).

The majority of recent studies on miRNAs are primarily focusing on the miRNAs of animals or plants, and there is a scarcity of research on fungal miRNAs. The miRNA-mediated post-transcriptional regulation of genes is a novel gene regulation strategy that is used to regulate the expression of protein-coding genes by targeting mRNAs *via* cleavage or translational repression. Our current understanding of target recognition by miRNAs suggests that the mRNA sequence is complementary to bases 2–8 of miRNAs (referred to as the seed sequence) in the majority of miRNA-mediated silencing complexes (miRISCs). The seed region has the highest complementarity to the 3′-untranslated region (UTR) of the mRNA of the target gene, and previous studies have demonstrated that various miRNAs with numerous functions regulate multiple target genes *via* different mechanisms (Kiriga et al., 2020). The recent advancements in sequencing technologies and bioinformatics tools have facilitated the identification of milRNAs in various fungi (Zhou J. H. et al., 2012; Mu et al., 2015; Li et al., 2016). However, there is a scarcity of information regarding the functions and target recognition mechanism of fungal milRNAs.

Understanding the regulatory mechanisms of fungal milRNAs may aid in breeding novel varieties of edible mushroom. *Pleurotus cornucopiae* is one of the most extensively cultivated mushrooms in China, and has a high nutritional and medicinal value. The mechanism underlying the development of fruiting bodies is a complex process that is regulated by both genetics and environment, and has been a topic of immense interest in recent years. Numerous genes that play an important role in the development of mushrooms have been identified and characterized. For instance, it has been demonstrated that the genes that encode the FvHmg1 and LFC1 transcription factors negatively regulate the fruit body development of *Flammulina velutipes* (Wu et al., 2020; Meng et al., 2021). Additionally, genes encoding SsNox2 NADPH oxidases contribute to the generation of reactive oxygen species (ROS), which are essential for the sclerotia development of *Sclerotinia sclerotiorum* (Kim et al., 2011). Glutathione peroxidase (GPX), which aids in maintaining ROS homeostasis, has a complex influence on the growth of the filamentous fungi *Hypsizygus marmoreus* (Zhang J. J. et al., 2020). Additionally, the genes encoding protein kinases in the mitogen-activated protein kinase (MAPK) signaling pathway play an important role in cellular regulation in fungi by regulating phosphorylation and dephosphorylation. A previous study on *Metarhizium robertsii* revealed that the MAPK signaling cascade plays a regulatory role in the formation of conidia and stress tolerance (Chen et al., 2016). The SakA response factor of *Aspergillus nidulans* can transmit osmotic and oxidative stress signals to the MAPK signaling pathway and regulate the growth, development, and stress response of *A*. *nidulans* (Lara-Rojas et al., 2011). Another study reported that the adenosine cyclase of the cyclic adenosine monophosphate (cAMP) signal transduction pathway aids in the transformation of yeast morphology to mycelial morphology, and plays a crucial role in mycelial growth (Rocha et al., 2001).

Recent studies have demonstrated that small RNAs play vital roles in fungal development. For instance, it has been demonstrated that milR4 and milR16 mediate the development of fruiting bodies in *Cordyceps militaris*. The disruption of milR4 results in the non-formation of fruiting bodies while the disruption of milR16 results in the formation of abnormal fruiting bodies with pale yellow-colored primordia (Shao et al., 2019). In contrast, the overexpression of *Po-MilR-1* in *P*. *ostreatus* results in slow mycelial growth and formation of abnormal pilei with irregular edges (Xu et al., 2021). However, only one milRNA of *P*. *ostreatus* that plays a vital role in mycelial growth has been identified and purified to date. Therefore, the potential roles of milRNAs in the development of fruiting bodies of *P*. *cornucopiae* is poorly understood to date owing to the scarcity of information, and further studies are necessary in this regard.

In this study, the genes encoding the Dicer, argonaute (AGO), and RNA-dependent RNA polymerase (RDRP) proteins of *P*. *cornucopiae* were identified and their expression profiles were determined at different developmental stages. The milRNAs related to the development of *P*. *cornucopiae* were determined by small RNA sequencing and *in silico* analyses. The potential targets of the milRNAs in the genome of *P*. *cornucopiae* were additionally detected, and the expression and functions of these target genes were determined by transcriptome sequencing and bioinformatics analyses. The theoretically predicted milRNAs and their gene targets were experimentally validated by quantitative real-time PCR (qRT-PCR) and dual-luciferase activity assay. Finally, one milRNA of *P. cornucopiae* was identified, and its functions in the development of fruiting bodies of *P. cornucopiae* were determined by overexpression and silencing. The results are anticipated to provide a foundation for research on milRNA function and the application of milRNAs in the development of edible mushrooms.

## 2. Materials and methods

### 2.1. Strains and media

The CCMSSC 00406 strain of *P. cornucopiae* was obtained from the China Center for Mushroom Spawn Standards and Control. The fungal mycelia were inoculated on potato dextrose agar (PDA) at 28°C for 6 days. The cottonseed hull culture medium was used to the mushroom production experiment according to our previous study (Qiu et al., 2018). The samples of mycelia, primordia, and caps of fruiting bodies, denoted as M, P, and C, were collected and stored at −80°C after freezing with liquid nitrogen.

### 2.2. Identification of RDRP, Dicer, and AGO proteins

The amino acid sequences of the proteins which were functionally annotated as RDRP, Dicer, and AGO proteins were derived from the genome of *P. cornucopiae*. The sequences were subjected to domain analyses using the conserved domain database of NCBI for determining protein function. The sequences of the RDRP, Dicer, and AGO proteins of other fungi were retrieved from GenBank, and aligned to the corresponding proteins of *P. cornucopiae* with CLUSTALW (Thompson et al., 1994; Hu et al., 2013; Wang et al., 2021). A phylogenetic tree was constructed using the maximum likelihood method based on the Tamura-Nei model and 1,000 bootstrap replicates with the MEGA software, version 5.0 (Kumar et al., 2016) for analyzing the relationships between the RDRP, Dicer, and AGO proteins of *P. cornucopiae* and those of other fungi in literature.

### 2.3. Deep sequencing of mRNAs and small RNAs

Comparative mRNA and milRNA analyses were performed using the CCMSSC 00406 strain of *P. cornucopiae*. Samples of the different developmental stages, including M, P, and C, of CCMSSC 00406 were collected in three biological replicates and subjected to mRNA and small RNA sequencing. The total RNA was extracted from all the samples using TRIzol reagent (Invitrogen, Carlsbad, United States), according to the manufacturer’s instructions, and subsequently treated with RNase-free DNase I (TaKaRa, Shiga, Japan) for removing the genomic DNA. The concentration of the RNA was evaluated using a NanoDrop 2000 spectrophotometer (ThermoFisher, Waltham, United States), and the integrity of the RNA was detected using an Agilent 2100 Bioanalyzer (Agilent, Palo Alto, United States). The cDNA libraries were constructed according to the protocol for library construction and sequenced on an Illumina NovaSeq 6000 platform (Illumina, San Diego, United States). The small RNAs were isolated from the total RNA by polyacrylamide gel electrophoresis (PAGE) with a 6% Tris, boric acid, and EDTA (TBE)—urea denaturing gel, and ligated to specific 5′ and 3′ adaptors. The cDNA libraries were sequenced on an Illumina HiSeq2500 platform following reverse transcription and appropriate amplification and purification.

### 2.4. Bioinformatics analyses of mRNAs and small RNAs

The clean data (clean reads) were obtained from the raw RNA-seq data by removing the reads containing adapters, poly-N, and low quality reads. The HISAT2 software was used for mapping the reads to the reference genome.^1^ The genes were annotated by BLAST search against using the NCBI non-redundant protein sequence (NR), Gene Ontology (GO; Tatusov et al., 2000), Kyoto Encyclopedia of Genes and Genomes (KEGG; Kanehisa et al., 2004), KOG (Koonin et al., 2004), and protein family (Pfam) databases. The expression levels of the genes were estimated by fragments per kilobase of transcript per million fragments mapped (FPKM). The genes that were differentially expressed among the different developmental stages were determined using the DESeq2 tool, with a false discovery rate (FDR) of <0.05. The differentially expressed genes (DEGs) were subjected to GO and KEGG pathway enrichment analyses, and the 20 most enriched pathways with the lowest Q values were selected (Mao et al., 2005).

The high-quality small RNA sequence reads (clean reads) were filtered from the total reads by removing the low-quality reads, reads containing adaptor sequences, and sequences smaller than 18 nt or longer than 30 nt. The clean reads were subsequently aligned to the genome of *P. cornucopiae* using the Bowtie software (Langmead et al., 2009). The different non-coding RNAs, including rRNAs, snRNAs, tRNAs, and snoRNAs, were identified using the Bowtie software, and subsequently removed. The remaining unannotated small RNAs were analyzed for detecting the known miRNAs from miRBase, and the miRDeep2 tool was used for predicting the novel milRNAs (Friedlander et al., 2012). The Randfold software was used for predicting the secondary structures of the novel milRNAs. The expression of the milRNAs in different developmental stages was calculated using the transcripts per million (TPM) normalization method (Fahlgren et al., 2007). The milRNAs that were differentially expressed in the M, P, and C at the different developmental stages were identified using the DESeq tool (Zeng et al., 2018). The target genes of the milRNAs were predicted based on the milRNA sequence information using the TargetFinder,^2^ miRanda (Enright et al., 2003), and RNAhybrid (Rehmsmeier et al., 2004) webtools, as previously described.

### 2.5. Analysis of the expression of milRNAs and mRNAs related to the development of fruiting bodies

The expression levels of the milRNAs were quantified by stem-loop real-time PCR, using 5S rRNA as the internal control for each sample (Zhou Q. et al., 2012). The stem-loop primers in the reverse transcription kit and the upstream primers used for qRT-PCR were designed using miRNA design software.^3^ The first-strand cDNA was synthesized using a miRNA first Strand cDNA Synthesis Kit (Vazyme, Nanjing, China), according to the manufacturer’s instructions. The miRNA Universal SYBR qPCR Master Mix (Vazyme, Nanjing, China) and an ABI 7500 real-time PCR amplifier (Applied Biosystems, Foster City, CA, United States) were used for qRT-PCR, as described in our previous study. The expression of the target genes of the milRNAs was detected using glyceraldehyde-3-phosphate dehydrogenase (GAPDH) as the internal control for each sample, as previously described (Hou et al., 2021). The relative expression levels of the milRNAs and their target genes in the different stages were quantified using the comparative threshold cycle (CT) 2^−△△CT^ method. The primers used for qRT-PCR amplification of the milRNAs are enlisted in Supplementary Table S1.

### 2.6. Dual-luciferase activity assay

A ~ 200 bp sequence near the binding site of the wild-type (WT) *g8971* and mutant *g8971* genes were synthesized and inserted into the pmirGLO vector. Briefly, HEK293T cells were co-transfected with 0.2 μg of the luciferase reporter vector (g8971-WT or g8971-MT) and 10 ng of milR-20-mimic or mimic NC together with the renilla luciferase construct using lipofectamine TM 2000 (Invitrogen), according to the manufacturer’s instructions (Grentzmann et al., 1998). The HEK293T cells were collected 48 h post-transfection, and the activities of luciferase and renilla luciferase were measured using a Dual-Luciferase® Reporter Assay System (Promega, Wisconsin, United States) according to the instructions provided (Cai et al., 2017). Five biological repetitions of the experiment were averaged and analyzed using Student’s *t* test.

### 2.7. Overexpression and silencing of milR20

For the overexpression of milR20, a precursor of milR20 was amplified using the WT genomic DNA as the template, and inserted by homologous recombination into a pCAMBIA1300 vector containing the gpd promoter of *P. ostreatus*. MilR20 was silenced using the short tandem target mimic (STTM) technology; STTM contains two target mimic (TM) sequences and a 48 nt-long specific linker sequence. The TM sequence corresponds to the sequence that is complementary to milR20 and possesses a tri-nucleotide that is inserted between the 10 and 11th bases of milR20. The STTM sequence was subsequently ligated to the pCAMBIA1300 vector.

All the recombinant plasmids were verified by sequencing and transfected into WT cells by *Agrobacterium tumefaciens*-mediated transformation (ATMT), as previously described. The strains in which milR20 was overexpressed and silenced were detected by PCR for cloning the *hpt* gene. The expression levels of milR20 following overexpression and silencing were quantified by qRT-PCR. The diameters of the colonies of the WT strain and the strains in which milR20 was overexpressed (OE-milR20) and silenced (STTM-milR20) were measured using the cross method for determining the mycelial growth rate. The WT, OE-milR20, and STTM-milR20 strains were separately inoculated on a culture medium for analyzing the primordial formation time, the developmental cycle of the fruiting bodies, and the spore print.

### 2.8. Statistical analysis

All statistical analyses were performed using the SPSS 26.0 software (SPSS Inc., Chicago, United States). The data are presented as mean ± SEM values. Statistical significance was defined as ^*^(*p* < 0.05), ^**^(*p* < 0.01), and ^***^(*p* < 0.001). The GraphPad Prism 8.0.1 software (GraphPad Software Inc., San Diego, United States) and Excel 2010 software (Microsoft, Redmond, WA, SA, United States) were used for drawing figure.

## 3. Results

### 3.1. mRNA sequencing and analyses

The expression profiles of the genes expressed in the M, P, and C stages across the three different developmental stages were determined using mRNA-seq. Three biological replicates were sequenced for each tissue type and a total of approximately 428 million clean reads were obtained from all the samples after filtering the low-quality reads. The number of reads in the samples ranged from 39 to 65 million. The reads were mapped to the genome of *P. cornucopiae*; approximately 32–53 million reads (80–82% of the total reads) mapped to the genome of *P. cornucopiae* (Supplementary Table S2). The Pearson correlation coefficients results indicated that the reproducibility between biological replicates was high enough for subsequent studies (Supplementary Figure S1).

**Figure 1:**
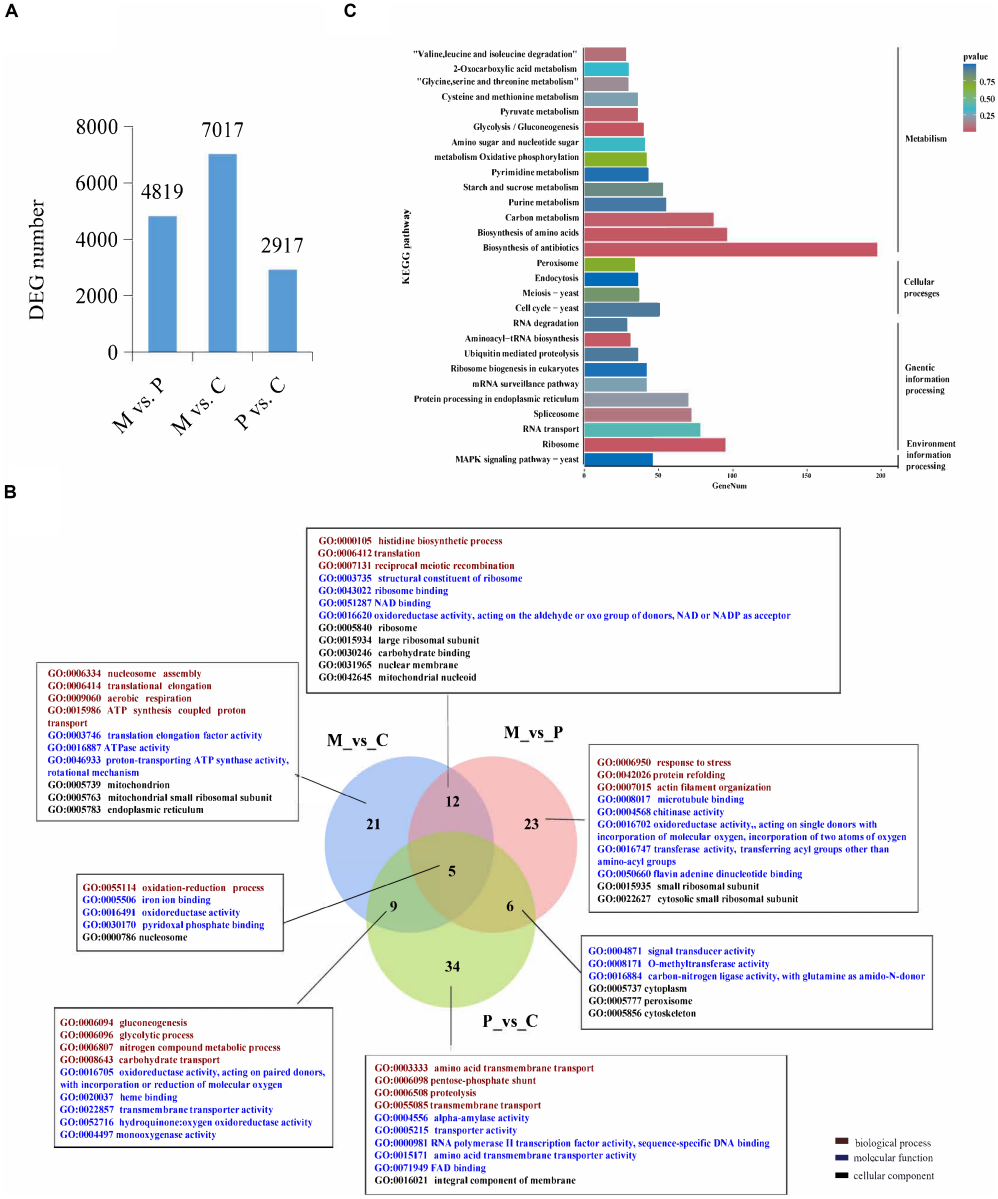
Analysis of the differentially expressed genes (DEGs) across the different developmental stages. **(A)** The number of DEGs among the different developmental stages. **(B)** Venn diagram depicting the GO term that the DEGs enriched in all three comparative groups. GO terms in figure are all significantly enriched terms with *p* < 0.05. **(C)** KEGG enrichment analyses of the DEGs among the different developmental stages.

In order to identify the genes that are involved in the development of *P. cornucopiae*, the DEGs among the different developmental stages were identified using the following criteria: FDR ≤ 0.05 and fold change (FC) ≥ 1.5. The number of DEGs in the M vs. P, M vs. C, and P vs. C comparison groups was determined to be 4,819, 7,017, and 2,917, respectively (Figure 1A). A total of 7,934 DEGs were identified from all the comparison groups, of which the number of DEGs in the M vs. C comparison group was highest. This indicated that the number of genes differentially expressed between the different intervals was higher than that between adjacent stages, and this finding was consistent with the organizational difference.

The DEGs were subjected to GO and KEGG pathway enrichment analyses. The results of GO enrichment analysis revealed that the DEGs in the M vs. C, P vs. C, and M vs. P comparison were significantly enriched in 46, 54, and 47 GO terms (*p* < 0.05), respectively. The significantly GO terms in the overlapped groups and the top 10 most significantly enriched GO terms in a single group were present. In biological process, the significantly enriched GO terms shared in M vs. C and P vs. C were gluconeogenesis, glycolytic, nitrogen compound metabolic process, and carbohydrate transport. The significantly enriched GO terms shared in M vs. P and M vs. C were histidine biosynthetic process, translation, and reciprocal meiotic recombination. The significantly enriched GO terms shared in M vs. C, P vs. C and M vs. P were oxidation-reduction process. The significantly enriched GO terms in single group were translational elongation, response to stress, transmembrane transport etc. In molecular function, the significantly enriched GO terms in different group were ATPase activity, oxidoreductase activity, transporter activity, and signal transducer activity etc. In cellular component, the significantly enriched GO terms in different stage were mitochondrion, ribosome, intergral component of membrane etc. These findings indicated that the DEGs involved in energy metabolic process, signal transduce process, and DEGs located to mitochondrion and membrane could play a role in the development of fruiting bodies in *P. cornucopiae* (Figure 1B). The total 7,934 DEGs from all three comparison group were used for KEGG analysis and 20 most enriched KEGG pathways were present. The results revealed that the DEGs were enriched in the MAPK signaling pathway, metabolism, cell cycle, and protein processing endoplasmic reticulum terms (Figure 1C). These findings indicated that the DEGs that were involved in these pathways could play a key role in the development of *P. cornucopiae*.

### 3.2. Identification and analysis of the genes involved in milRNA biogenesis and function

**Figure 2:**
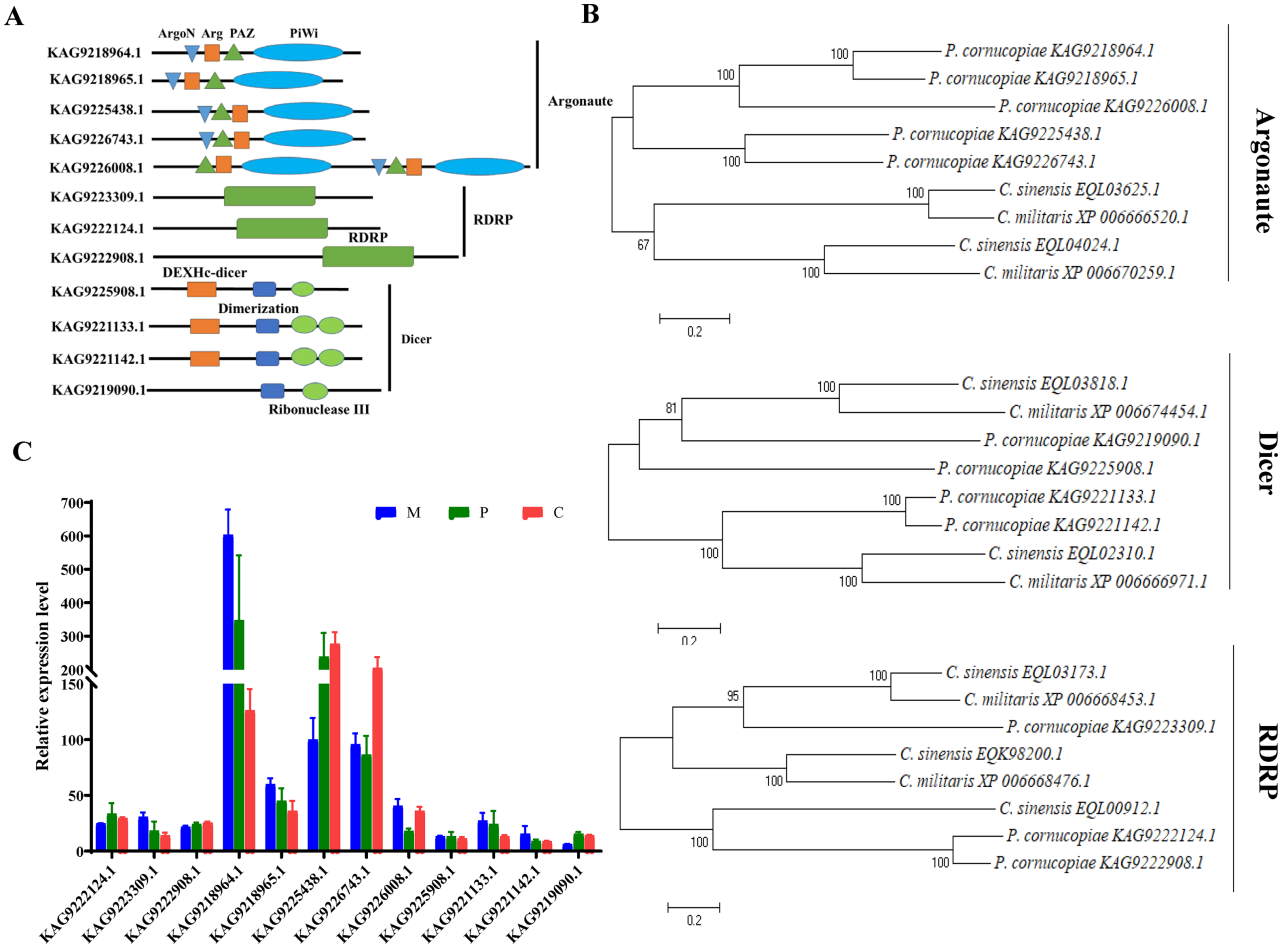
Analysis of the Dicer, RDRP, and AGO proteins of *Pleurotus cornucopiae* and other fungi. **(A)** Conserved domain analysis of the Dicer, RDRP, and AGO proteins of *P. cornucopiae*. **(B)** Phylogenetic analysis of the Dicer, RDRP, and AGO proteins of *P. cornucopiae* and other fungi. **(C)** Expression levels of the genes determined from the transcriptome data during the different developmental stages of *P. cornucopiae*. The data are expressed as the mean ± standard error (SE) of the data obtained from three replicates.

RNA-dependent RNA polymerase, AGO, and Dicer proteins play a key role in milRNA biogenesis and function in eukaryotes. Therefore, the presence of these proteins could indicate that *P. cornucopiae* contains miRNAs. Therefore, the genes encoding RDRP, AGO, and Dicer proteins were identified in the genome of *P. cornucopiae* by BLASTp. A total of five homologs of AGO (KAG9218964.1, KAG9218965.1, KAG9225438.1, KAG9226743.1, and KAG9226008.1), three homologs of RDRP (KAG9223309.1, KAG9222124.1, and KAG9222908.1), and four homologs of Dicer (KAG9225908.1, KAG9221133.1, KAG9221142.1, and KAG9219090.1), which shared the best sequence homology with the genome sequence of *P. cornucopiae*, were identified. In order to ensure that these proteins were indeed homologs of Dicer, AGO, and RDRP proteins, the conserved domains in these predicted protein sequences were predicted by searching against the NCBI database. The results demonstrated that all the five homologs of AGO contained the N-terminal domain (ArgoN), Argonaute Linker 1 domain (Arg), Piwi AGO and Zwille (PAZ), and Piwi domains. The three homologs of RDRP contained the RDRP domain, while the four homologs of Dicer contained the ribonuclease III domain (Ribonuclease III) and a conserved Dicer dimerization domain (Figure 2A). The results of phylogenetic analysis demonstrated that the sequences of the RDRP, AGO, and Dicer proteins of *P. cornucopiae* were highly homologous to those of *C. sinensis* and *C*. *militaris* (Figure 2B). These findings suggested that *P. cornucopiae* possesses functional milRNA machinery.

In order to further explore the possible role of milRNAs in the developmental process of *P. cornucopiae*, the expression levels of RDRP, AGO, and Dicer at the different developmental stages were analyzed from the transcriptome data. The results demonstrated that the expression levels of AGO were higher than that of the other genes at each of the developmental stages. The expression levels of the genes encoding RDRP, AGO, and Dicer proteins varied across the different developmental stages of *P. cornucopiae* (Figure 2C), which suggested that the expression and functions of milRNAs were various during development.

### 3.3. Sequencing and analyses of the milRNAs in the different development stages

In order to identify the milRNAs that are related to the development of fruiting bodies in *P. cornucopiae*, the small RNAs in the M, P, and C stages were subjected to sequencing, which was performed in triplicate. The samples of M tissues were denoted as M-1, M-2, and M-3, while the samples of P tissues were denoted as P-1, P-2, and P-3, and the samples of C tissues were denoted as C-1, C-2, and C-3. Approximately 20 million raw reads were obtained from each sample. Approximately 10 million clean reads with lengths varying between 18 and 30 nt were obtained after filtering the low-quality reads and trimming the 3′-specific adaptors, and the remaining small RNAs were annotated. The clean reads were aligned to the genome of *P. cornucopiae*, and the results demonstrated that the 2–6 million reads included various small ncRNAs, including rRNAs, tRNAs, and snoRNAs, which accounted for 21.35–60.19% of the total clean reads obtained from the different developmental stages. The 4–8 million unannotated clean reads were subsequently analyzed for further prediction of milRNAs, which accounted for 39.64–77.94% of the total clean reads obtained from the different developmental stages (Supplementary Table S3).

**Figure 3:**
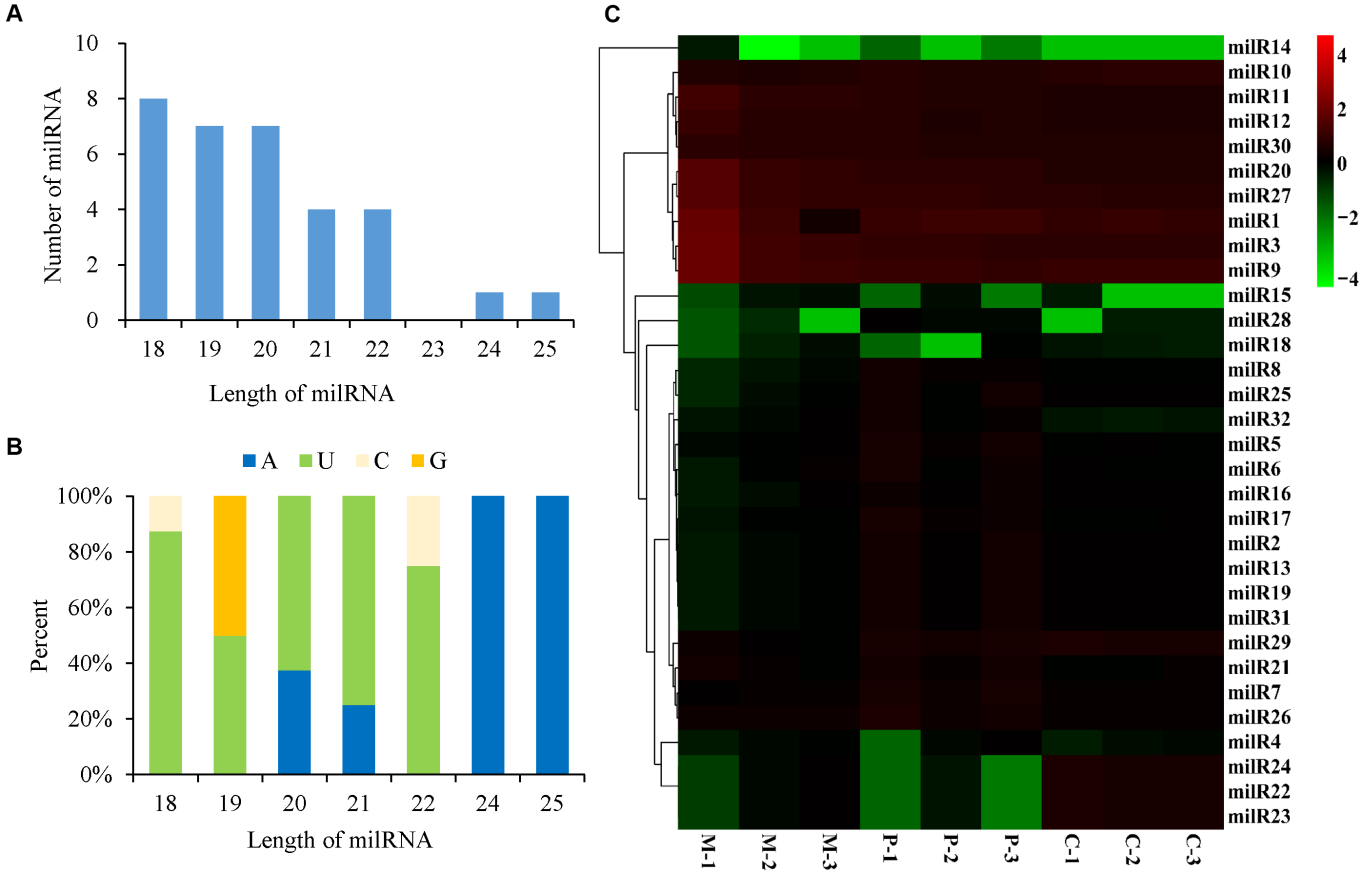
Characteristics and expression levels of the milRNAs in the three developmental stages of *Pleurotus cornucopiae*. **(A)** Length distribution of the milRNAs of *P. cornucopiae.* **(B)** 5′-terminal nucleotide bias of the milRNAs of *P. cornucopiae.* **(C)** Heatmap depicting the expression levels of the milRNAs in the three development stages.

A total of 32 milRNAs were finally identified from the different developmental stages of *P. cornucopiae*, and 31, 26, and 30 milRNAs were identified from the M, P, and C stages, respectively (Supplementary Figure S2). The milRNAs were denoted as milR1–milR32 (Supplementary Table S4). Analysis of the length distribution of the milRNAs revealed that the lengths of the mature milRNAs ranged from 18 to 25 nt. The majority of these milRNAs were 18–22 nt long, and accounted for 93.75% of the total mature milRNAs, which was higher than the number of milRNAs with other lengths (Figure 3A). The results of nucleotide bias analysis revealed that the nucleotides at the 5′-terminus had a strong preference for uracil (U) in the milRNAs that were 18–22 nt long, which was similar to that observed in animals and plants. However, the milRNAs that were 24–25 nt long were enriched in adenine (A) at the 5′-end (Figure 3B).

The abundance of milRNAs was normalized according to the TPM normalization method. Of the 32 milRNAs, 25 were expressed in all the three developmental stages, while the other seven milRNAs were expressed in one or two of the developmental stages (Supplementary Figure S2). The heatmap in Figure 3C demonstrates that the expression patterns of the milRNAs varied across the different development stages and nine of these milRNAs were highly expressed during the entire development of *P. cornucopiae*. The findings suggested that the milRNAs that were expressed at high levels in all the three developmental stages could play a crucial role in the development of *P. cornucopiae*.

**Figure 4:**
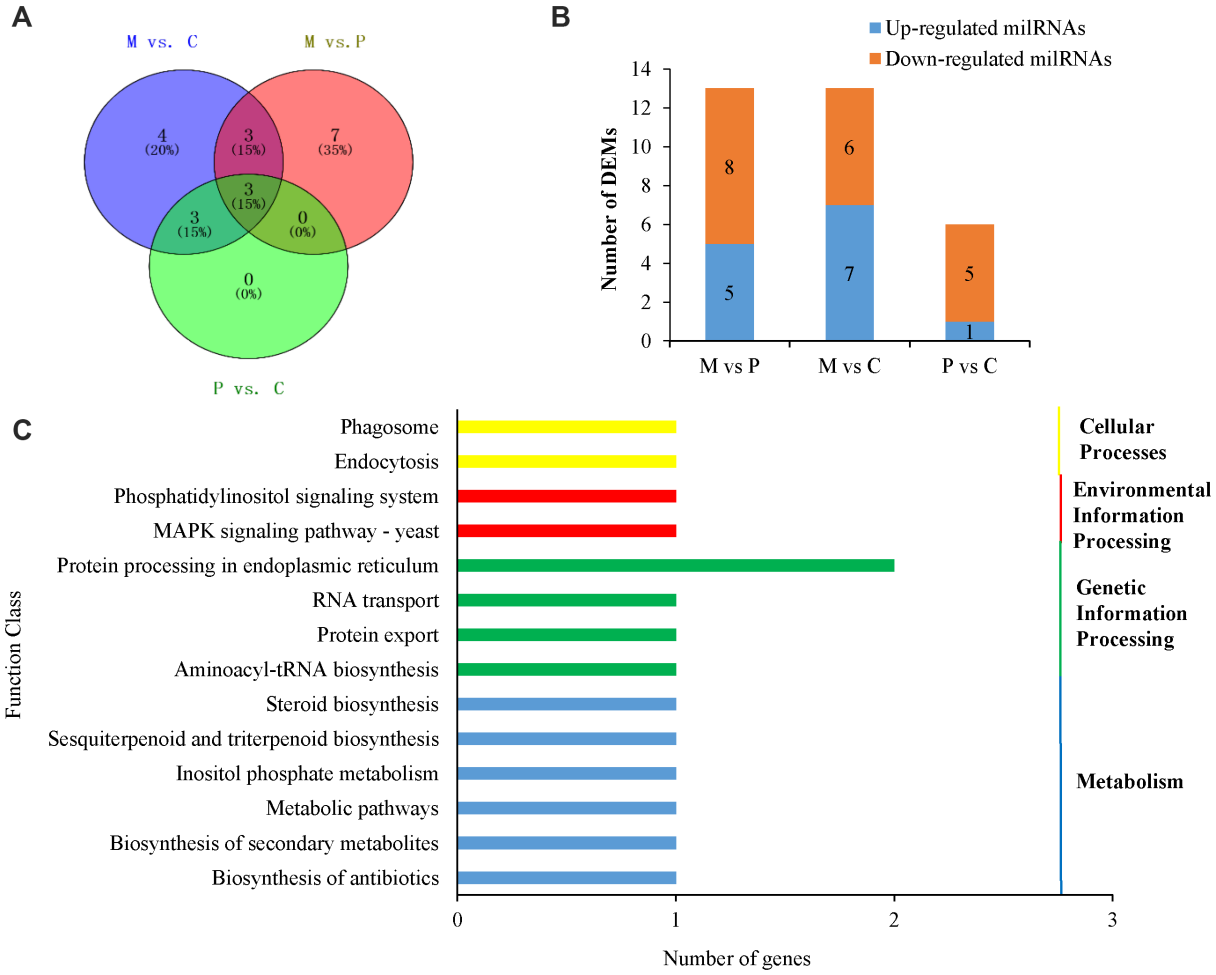
Analyses of DEMs. **(A)** Venn diagram depicting the DEMs among the different developmental stages. **(B)** Expression of DEMs in the different comparison groups. **(C)** Results of KEGG pathway analysis of the target genes of DEMs.

To explore the milRNAs that are related to the development of fruiting bodies in *P. cornucopiae*, we analyzed the differentially expressed milRNAs (DEMs) across the three developmental stages. *p* < 0.05 was regarded as the threshold for determining the significant differences in milRNA expression. The results demonstrated that 13, 13, and 6 milRNAs were significantly different expressed in the M vs. P, M vs. C, and P vs. C comparison groups, respectively. A total 20 DEMs were identified in the three comparison groups, of which three DEMs were common between the M vs. C and P vs. C comparison groups, and could play an important role in the development of fruiting bodies (Figure 4A). Analysis of the expression levels of the DEMs in the different comparison groups revealed that the number of DEMs downregulated was higher than that of upregulated in the C vs. P comparison group, while the number of upregulated DEMs was approximately equal to that of the downregulated DEMs in the M vs. P and P vs. C comparison group (Supplementary Table S5; Figure 4B). These results indicated that the DEMs that were downregulated along the development of the fruiting body could play a more crucial role in the development of *P. cornucopiae*.

In order to elucidate the potential functions of the DEMs in the development of *P. cornucopiae*, the target genes of milRNAs were predicted using the TargetFinder, miRanda, and RNAhybrid software, as previously described. A total 17 milRNAs in the 20 DEMs were predicted to target 44 genes. The findings revealed that some of the DEMs could regulate several target genes and more than one milRNA could regulate the same target gene (Supplementary Table S5), which was consistent with the reports of previous studies on plant and animal miRNAs. These target genes were subjected to functional enrichment analyses using the GO and KEGG databases. The findings demonstrated that 41 of the target genes of DEMs were functionally annotated. The results of GO enrichment analysis revealed that the target genes were primarily enriched in the transporter activity, receptor activity, and catalytic activity terms in the molecular function category; the signaling pathways, cellular processes, and response to stimulus terms in the biological process category; and the organelles and membranes terms in the cellular component category (Supplementary Figure S3). The results of KEGG enrichment analysis demonstrated that the target genes were enriched in different pathways, including the phosphatidylinositol signaling system, endocytosis, MAPK signaling pathway, protein processing in endoplasmic reticulum, and other metabolic pathways (Figure 4C).

### 3.4. Correlation analysis of milRNAs and mRNAs

**Figure 5:**
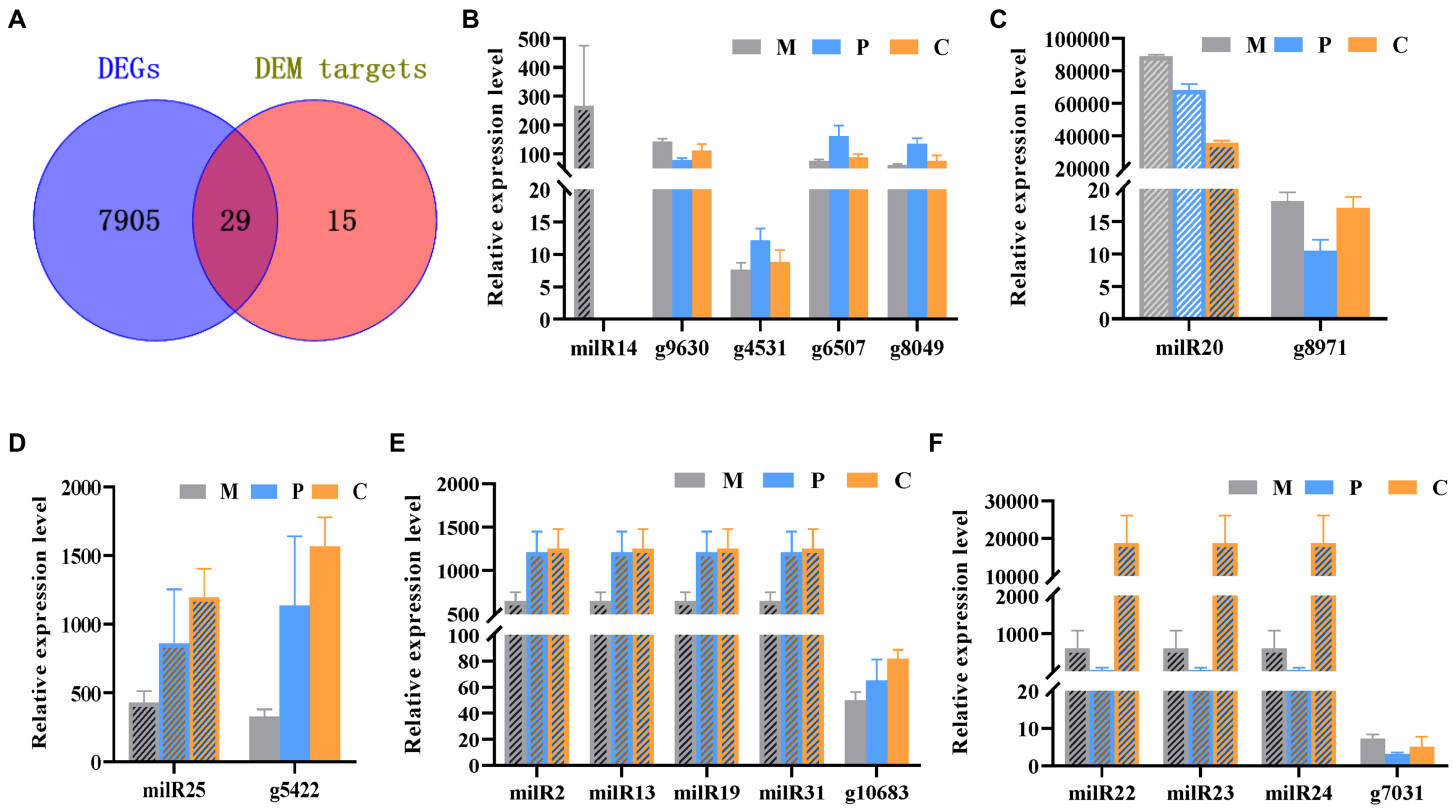
Integrated analyses of mRNA and milRNA data. **(A)** Venn diagram depicting the DEGs in the different stages of development and DEM targets. **(B–F)** Analysis of the expression levels of the DEMs and their targets DEGs.

The Venn diagram depicting the DEGs and the target genes of DEMs indicated that 29 of the 44 target genes of the DEMs were differentially expressed across the different developmental stages (Figure 5A). Of these, only seven DEGs could be mapped by KEGG pathway analyses, and were found to be enriched in the MAPK signaling pathway, phosphatidylinositol signaling system, protein processing in endoplasmic reticulum, and other metabolic pathways that may play crucial roles in the growth and development of *P. cornucopiae* (Table 1). For instance, the findings revealed that the *g4622* gene was involved in the phosphatidylinositol signaling system, *g9630* was enriched in endocytosis, *g8971* was enriched in the MAPK signaling pathway, and the remaining genes were involved in other pathways.

**Table 1:** Enriched KEGG pathways of target DEGs of DEMs.

milRNA	Target	KEGG pathway	Target gene annotation
milR14	g9630	Endocytosis (ko04144)	Putative GTPase activating protein
milR14	g8049	RNA transport (ko03013)	Eukaryotic initiation factor 4E
milR14	g6507	Aminoacyl-tRNA biosynthesis (ko00970)	Arginine-tRNA ligase
milR14	g4531	Biosynthesis of antibiotics (ko01130)	Squalene/phytoene synthase
milR19	g10683	Protein processing in endoplasmic reticulum (ko04141)	Uncharacterized J domain-containing protein
milR20	g8971	MAPK signaling pathway (ko04011)	Pheromone A receptor
milR25	g5422	Protein processing in endoplasmic reticulum (ko04141)	Hsp70 protein

MiRNAs are important regulators of gene expression and act *via* degradation or translational repression of target mRNAs (Zdanowicz et al., 2009). Correlation analysis of expression profiles of milRNAs and their targets showed that only milR20 and milR14 had the relative opposite expression trend to their targets in partial development stage (Figures 5B,C), while other miRNA-target did not (Figures 5D–F). The results demonstrated that milR14 could play a minor role in the development of fruiting bodies because it was not expressed in the P and C stages. However, the stage-specific expression pattern of milR14 suggest it may have important function during stage M development. However, the expression of milR20 was downregulated from the P to the C stage, while the expression of its target gene, *g8971*, was upregulated from the P to the C stage. These result indicated that *g8971* may be the target of milR20. milR20 was highly expressed in the three development stage with the expression level higher than 40,000, and it was a DEM from the M vs. C and P vs. C comparison group with the expression level decreased from M to C stage. The predict target of milR20, *g8971*, was DEGs at different developmental stages, and encodes a pheromone receptor that involved in the MAPK signal pathway which plays an important role in the development. These results therefore indicated that milR20 could negatively regulate the development of fruit bodies in *P. cornucopiae*.

In this study, qRT-PCR analysis was also performed for validating the expression profiles of the miRNA-target modules of interest, namely, milR20 and *g8971*. The expression pattern of milR20 and its target gene, *g8971*, obtained by RT-qPCR analysis was similar to that determined by high-throughput sequencing, and the findings revealed that the expression of milR20 tended to decrease from stages P to C (Supplementary Figure S4), while the expression level of *g8971* increased from stage P to C. The findings revealed that the milR20 had opposite expression trend to *g8971* from stage P to C. so milR20 was selected for further analyses.

### 3.5. milR20 targets *g8971* and inhibits its expression

**Figure 6:**
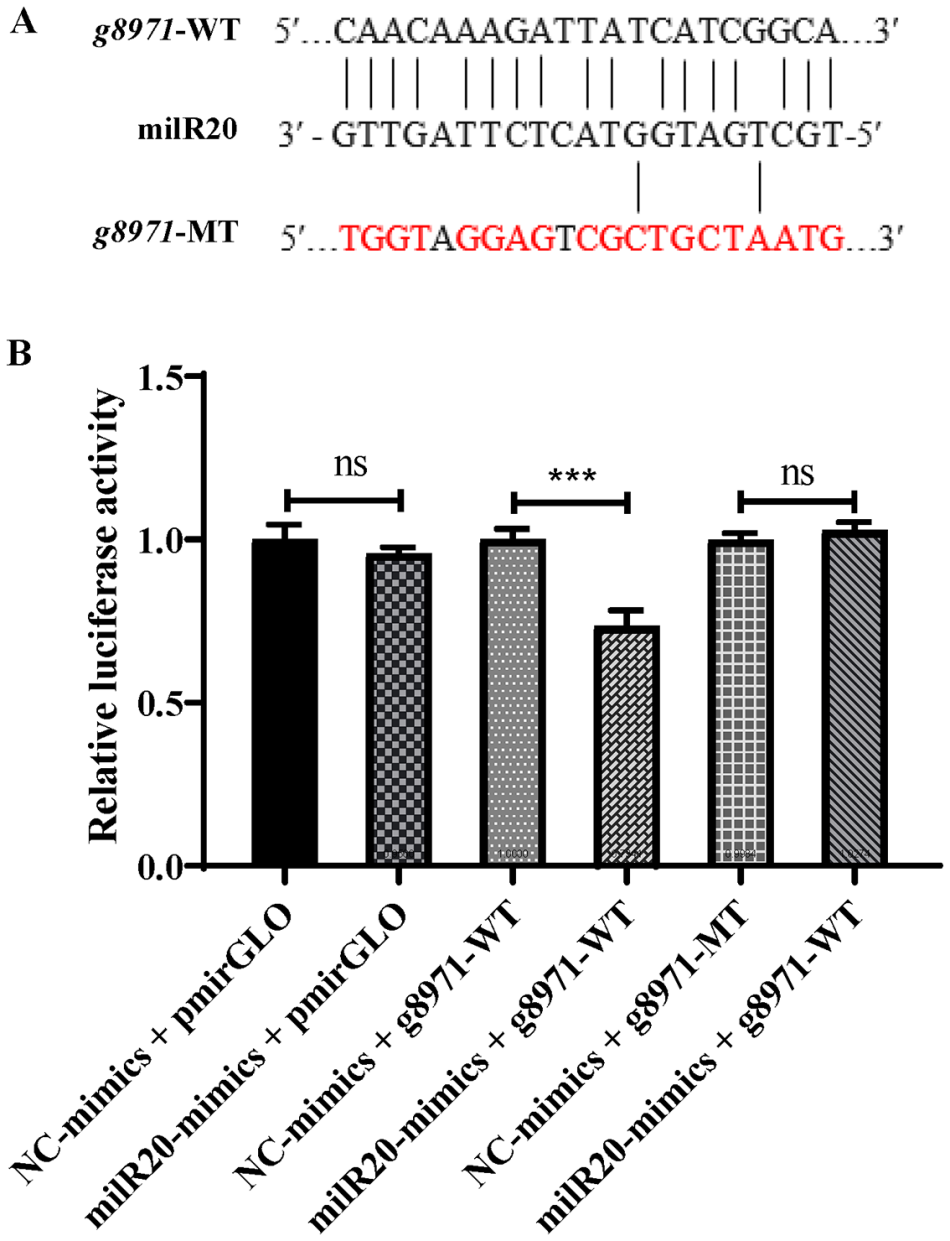
Verification of the targeting relationship between milR20 and *g8971*. **(A)** The specific binding sites of milR20 in the sequence of *g8971* are depicted, and the mutation sites were designed based on this sequence. The sequences in red represent the mutation sites. **(B)** The activity of luciferase was measured by dual-luciferase reporter assays. The activities of luciferase of g8971-WT decreased markedly in cells transfected with miR-milR20 compared to that of the control. The values are depicted as the mean ± SE; ^***^p < 0.001.

The results of prediction using the RNAhybrid software revealed that milR20 targeted the 848–868 nt region of the *g8971* gene (Figure 6A). Dual-luciferase reporter assays were performed for elucidating the targeting relationship between milR20 and its target gene, *g8971*. The WT *g8971* reporter vector (g8971-WT) and the mutant plasmids (g8971-MT) were constructed, and co-transfection experiments were performed. The results of the dual-luciferase assay demonstrated that milR20 significantly reduced the relative luciferase activity of g8971-WT. However, there was no significant effect on the relative luciferase activity of g8971-MT. These findings therefore indicated that milR20 could negatively regulate the expression of *g8791* by directly binding to and targeting *g8971* (Figure 6B).

### 3.6. Functional analysis of milR20 by STTM-mediated silencing and overexpression

In this study, the copy number of milR20 in the genome of *P. cornucopiae* was first determined by comparing the milRNA sequence with the genome using the BLASTn program. The results demonstrated that there was only one copy of milR20 in the genome. The precursor sequence of milR20 (pre-milR20) was identified and analyzed using the miRDeep2 software. A 250 bp-long sequence of pre-milR20 was obtained and represented as hairpin structures, which verified that milR20 was a real milRNA (Supplementary Figure S5).

**Figure 7:**
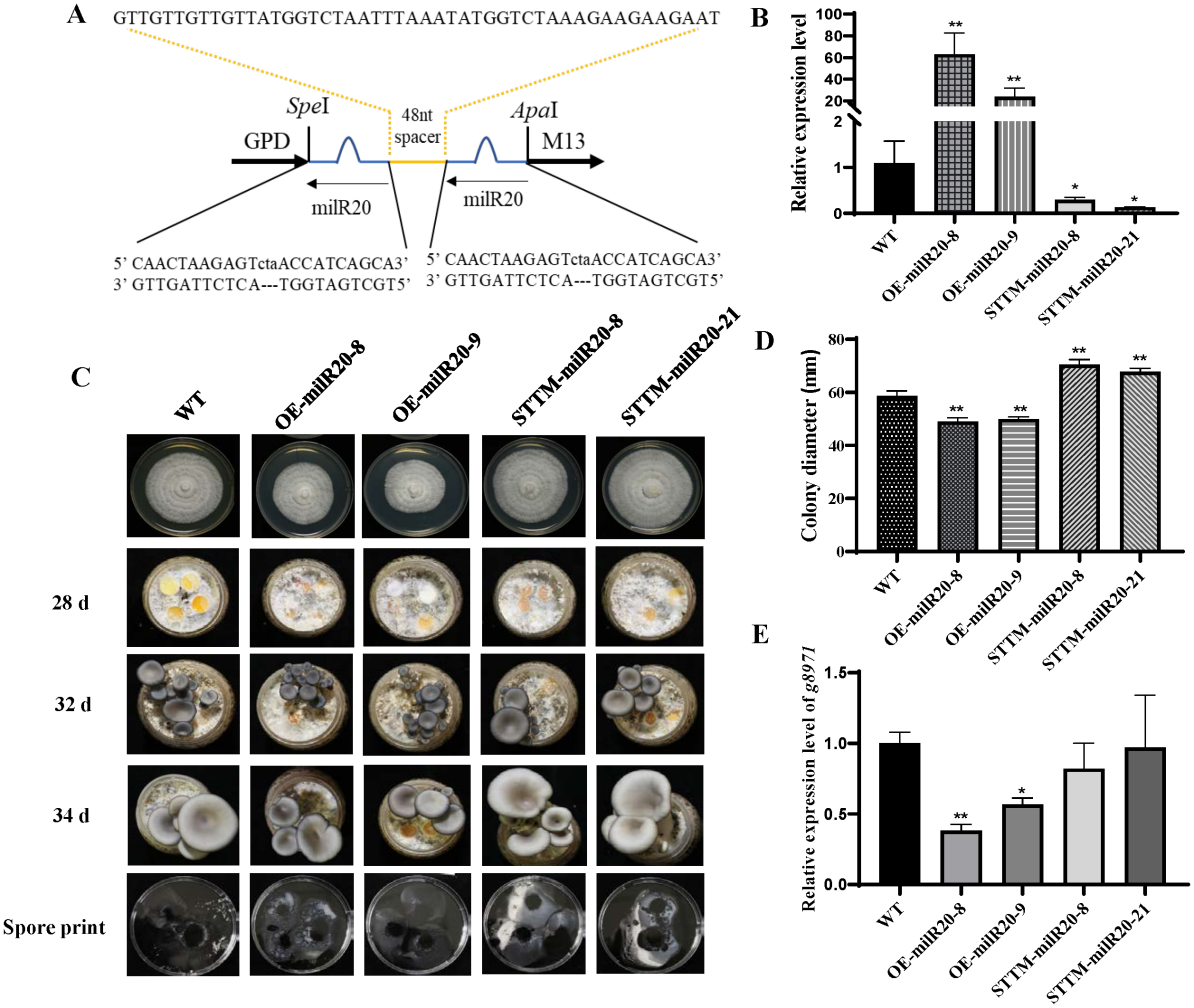
Effect of milR20 on the development of *Pleurotus cornucopiae*. **(A)** Schematic representation of the structure of the STTM plasmids that were used for silencing milR20. A target mimic with an unmodified central sequence (highlighted in blue) that was complementary to the central portion of milR20 and had a trinucleotide bulge was inserted at the cleavage site located at 10–11 nt of the miRNA. **(B)** Analysis of the relative expression levels of the WT and mutants by qRT-PCR. **(C)** Phenotype of the WT and mutant strains at different developmental stages. **(D)** Statistics of the colony diameter of the WT and milR20-recombinant strains under normal temperature, determined using SPSS. **(E)** Detection of the expression levels of the target *g8971* gene of milR20 in the WT and mutant strains by qRT-PCR; ^*^ and ^**^ indicate significant difference at *p* < 0.05 and *p* < 0.01, respectively.

In order to explore whether milR20 has any role in the development of *P. cornucopiae*, milR20 was separately silenced and overexpressed and the phenotypic effects were analyzed. STTM-mediated silencing has been shown to be an effective tool for inhibiting the activity of endogenous mature miRNAs in plants. In this study, we designed milR20 STTM constructs containing two same non-cleavable milRNA binding sites (highlighted in blue in Figure 7A), and linked by a 48–88 nt spacer (colored in yellow). We generated transgenic strains in which milR20 was overexpressed or silenced with STTM. Analysis of the expression levels of milR20 in the M stage of the transgenic strains revealed that the expression of milR20 increased significantly in the OE-milR20 strain compared to that in the WT, while its expression in the STTM-milR20 strain (32–75%) decreased significantly compared to that of the WT (Figure 7B).

The effect of milR20 on the development of *P. cornucopiae* was detected by analyzing the mycelial growth of the WT and mutant strains that had been incubated on PDA plates. The rate of mycelial growth in the OE-milR20 strain was significantly lower than that of the WT strain; however, the rate of mycelial growth in the STTM-milR20 strain was significantly higher than that of the WT strain (Figures 7C,D). These findings indicated that milR20 may play a negative role in mycelial growth.

The effect of milR20 on the development of the fruiting bodies of *P. cornucopiae* was subsequently detected by cultivating the mutant and WT strains on cultivation substrates. The time required for the formation of primordia was initially analyzed statistically. The primordia appeared on the 28th to the 29th day in all the strains, and there were no significant differences between the mutants and WT with respect to the time required for the formation of primordia. This finding indicated that milR20 had no influence on the formation of primordia (Supplementary Figure S6). Analysis of the morphology of the fruiting bodies revealed that the developmental cycle of the fruiting bodies was prolonged in the OE-milR20 strain that overexpressed milR20, compared to that of the WT. However, the developmental cycle of the fruiting bodies was slightly shortened in the STTM-milR20 strain compared to that of the WT. Analysis of the spore morphology revealed that the spore print density of the OE-milR20 strain did not exhibit significant alterations compared to that of the WT, while the quantity of spores produced by the STTM-milR20 strain increased slightly (Figure 7C). Detection of the expression levels of the target *g8971* gene of milR20 revealed that *g8971* was downregulated in the OE-milR20 strain, while there was no significant difference in the expression of *g8971* in the STTM-milR20 strain (Figure 7E). These results indicated that milR20 may negatively regulate the development of the fruiting bodies of *P. cornucopiae* by inhibiting the expression of *g8971*.

## 4. Discussion

Previous studies have demonstrated that miRNAs have an important role in the development of plant and animals. The development of high-throughput sequencing technologies has enabled the identification of milRNAs from various species of fungi in recent years. However, there is a scarcity of information regarding the functions of milRNAs in fungi. *Pleurotus cornucopiae* is an important mushroom that has been used for studying the functions of milRNAs in fungal development. AGO, Dicer, and RDRP proteins are key components of miRNA maturation and function in *N. crassa* and are conserved in *C*. *militaris* and other fungal species that have been reported to possess milRNAs (Lee et al., 2010; Yang et al., 2013; Shao et al., 2019; Wang et al., 2021). The present study demonstrated that the AGO, Dicer, and RDRP proteins of these fungal species were closely related to those of *P. cornucopiae*, which indicated that mechanisms of milRNA biogenesis also exist in *P. cornucopiae*. The number of genes encoding Dicer, AGO, and RDRP proteins vary across different fungal species, and the expression patterns of these homologs vary across the different developmental stages (Lee et al., 2010; Shao et al., 2019, 2020; Wang et al., 2021). This suggests that the homologs of genes encoding Dicers, AGOs, and RDRPs may play different roles during the formation of mature milRNAs from dsRNAs, and the expression and functions of milRNAs also vary during development. In this study, the expression level of genes encoding Dicers, AGOs, and RDRPs were analyzed by transcriptome analysis across the different developmental stages of *P. cornucopiae*. All the genes were expressed at different developmental stages, and there were variations in the expression patterns, which suggested that the homologs of genes encoding Dicers, AGOs, and RDRPs may function in a coordinated manner to regulate the expression and function of milRNAs during the development of *P. cornucopiae*.

The results of mRNA analysis demonstrated that the DEGs among the different development stages were mainly enriched in the signaling and growth terms in the biological process category of GO, and in the MAPK signaling pathway of KEGG. These findings indicated that the genes that were involved in the MAPK signaling pathway could be involved in the development of *P. cornucopiae*, and this finding was consistent with the results of previous studies on plants (Yi et al., 2016; Xiao et al., 2017; Chen et al., 2021). MAPK cascades are known to transmit extracellular signals to intracellular targets and play a crucial role in regulating several fundamental processes, including proliferation, differentiation, and cellular response to diverse extrinsic stresses (Guo et al., 2020).

In this study, the results of milRNA analysis demonstrated that a total of 32 milRNAs were identified in the M, P, and C stages, and the majority of these milRNAs were expressed at all the stages. However, the expression levels of the milRNAs varied across the three developmental stages. A total of 20 milRNAs were differently expressed in the three M vs. P, M vs. C, and P vs. C comparison groups, of which the number of downregulated DEMs was higher than the number of upregulated DEMs. These results indicated that the downregulated milRNAs could play a vital role in the development of *P. cornucopiae*. MiRNAs are important regulators of gene expression and function *via* degradation or translational repression of the target mRNAs (Moran et al., 2017). Integrated analysis of the milRNAs and mRNAs revealed that the target DEGs of the DEMs mapped to the MAPK signaling pathway, and this finding was consistent with the results of mRNA analysis. These results indicated that the milRNAs which regulated the MAPK signaling pathway could play a significant role in the development of *P. cornucopiae*, and was consistent with the findings of previous studies which reported that miRNAs regulate the activity of the MAPK cascade and influence cellular proliferation in animals (Chen et al., 2017; Xiao et al., 2018; Xu et al., 2018; Safa et al., 2020; Zhang H. et al., 2020).

Correlation analysis of the milRNA expression profiles and their target genes revealed that only a small number of the milRNA-mRNA pairs exhibited an opposite expression pattern in the different developmental stages. These results indicated that the expression of the majority of target genes was possibly not negatively regulated by the milRNAs. Previous studies have also demonstrated an incoherent regulation between miRNAs/milRNAs and their target genes (Shao et al., 2019). This could be attributed to the fact that milRNAs primarily mediate gene regulation by repressing mRNA translation in fungi and not *via* mRNA degradation. Considering the complex regulatory network of gene expression, this could be alternatively explained by the fact that the expression of target genes can also be regulated by other genes, including genes encoding transcription factors, and competing endogenous RNAs that competitively bind to miRNAs (Kartha and Subramanian, 2014).

In this study, the results of the dual-luciferase activity assay and qRT-PCR results revealed that milR20 targeted the *g8971* and inhibited the expression of *g8971*. The expression of milR20 was downregulated in both the M vs. C and P vs. C comparison groups, and the target gene of milR20, *g8971*, was invovled in the MAPK signaling pathway and could play a vital role in the development of *P. cornucopiae*. Therefore, the functions of milR20 in the development of *P. cornucopiae* were subsequently analyzed using overexpressing and silencing by short tandem target mimic (STTM) technology in this study.

The STTM technology mimics the binding of target miRNAs to RNA-induced silencing complex (RISC) to inhibit the functions of target miRNAs (Teotia et al., 2016). This method has been proven to be an effective and stable tool for blocking the activity of endogenous mature miRNAs in plants (Jia et al., 2015; Zhang et al., 2017). In this study, the expression levels of milR20 decreased significantly in the STTM-milR20 strain, indicating that the STTM technology can also be used to effectively silence fungal milRNAs. The mycelial growth rates and the development of fruit body in the OE-milR20 strain were reduced and prolonged, while those of the STTM-milR20 strain were the opposite in comparison to that of the WT strain. The expression level of *g8971* in the OE-milR20 were significantly decreased, while no significant difference in STTM-miR20 strains. These could be explained by the expression fold change difference of milR20 in different strains or the regulation of *g8971* by other genes. These findings indicated that milR20 could negatively regulate the growth of *P. cornucopiae* by repressing the expression of *g8971* regulating the MAPK signaling pathway.

## Data availability statement

The original sequence data of transcriptome can be found at the following link: https://www.ncbi.nlm.nih.gov/bioproject/PRJNA943625 and the milRNA sequence data can be found at the following link: https://www.ncbi.nlm.nih.gov/bioproject/PRJNA944818. Other data presented in this study are available in Supplementary material

## Author contributions

LZ designed the study and prepared the manuscript. YQ performed the experiments and analyzed the data. YQ, CH, MZ, XW, GL, YZ, and LZ discussed the results. All authors contributed to the article and approved the submitted version.

## Funding

This study was financially supported by the China Agriculture Research System (CARS20), Fundamental Research Funds for Central Nonprofit Scientific Institution (No. 1610132020004), National Key R&D Program of China (2022YFD1200600), and the Beijing Agriculture Innovation Consortium (BAIC03).

## Conflict of interest

The authors declare that the research was conducted in the absence of any commercial or financial relationships that could be construed as a potential conflict of interest.

## Publisher’s note

All claims expressed in this article are solely those of the authors and do not necessarily represent those of their affiliated organizations, or those of the publisher, the editors and the reviewers. Any product that may be evaluated in this article, or claim that may be made by its manufacturer, is not guaranteed or endorsed by the publisher.

## Supplementary material

The Supplementary material for this article can be found online at: https://www.frontiersin.org/articles/10.3389/fmicb.2023.1177820/full#supplementary-material

## Footnotes

^1^ https://www.ncbi.nlm.nih.gov/nuccore/WQMT00000000.2/

^2^ http://targetfinder.org

^3^ https://www.vazyme.com

## References

[ref1] Cai B. L. Li Z. H. Ma M. T. Wang Z. J. Han P. G. Abdalla B. A.. (2017). LncRNA-Six1 encodes a micropeptide to activate Six1 in cis and is involved in cell proliferation and muscle growth. Front. Physiol. 8:230. doi: 10.3389/fphys.2017.00230, PMID: 28473774 PMC5397475

[ref2] Chen J. Wang L. Yuan M. (2021). Update on the roles of rice MAPK cascades. Int. J. Mol.Sci. 22:1679. doi: 10.3390/ijms22041679, PMID: 33562367 PMC7914530

[ref3] Chen P. Xu W. Luo Y. Zhang Y. He Y. Yang S.. (2017). MicroRNA 543 suppresses breast cancer cell proliferation, blocks cell cycle and induces cell apoptosis via direct targeting of ERK/MAPK. Onco. Targets. Ther. 10, 1423–1431. doi: 10.2147/OTT.S118366, PMID: 28331335 PMC5348068

[ref4] Chen X. X. Xu C. Qian Y. Liu R. Zhang Q. Q. Zeng G. H.. (2016). MAPK cascade-mediated regulation of pathogenicity, conidiation and tolerance to abiotic stresses in the entomopathogenic fungus *Metarhizium robertsii*. Environ. Microbiol. 18, 1048–1062. doi: 10.1111/1462-2920.13198, PMID: 26714892

[ref5] Enright A. J. John B. Gaul U. Tuschl T. Sander C. Marks D. S. (2003). MicroRNA targets in *Drosophila*. Genome Biol. 5:R1. doi: 10.1186/gb-2003-5-1-r1, PMID: 14709173 PMC395733

[ref6] Fahlgren N. Howell M. D. Kasschau K. D. Chapman E. J. Sullivan C. M. Cumbie J. S.. (2007). High-throughput sequencing of *Arabidopsis* microRNAs: evidence for frequent birth and death of MIRNA genes. PLoS One 2:e219. doi: 10.1371/journal.pone.0000219, PMID: 17299599 PMC1790633

[ref7] Friedlander M. R. Mackowiak S. D. Li N. Chen W. Rajewsky N. (2012). miRDeep2 accurately identifies known and hundreds of novel microRNA genes in seven animal clades. Nucleic Acids Res. 40, 37–52. doi: 10.1093/nar/gkr688, PMID: 21911355 PMC3245920

[ref8] Grentzmann G. Ingram J. A. Kelly P. J. Gesteland R. F. Atkins J. F. (1998). A dual-luciferase reporter system for studying recoding signals. RNA 4, 479–486. PMID: 9630253 PMC1369633

[ref9] Guo Y. J. Pan W. W. Liu S. B. Shen Z. F. Xu Y. Hu L. L. (2020). ERK/MAPK signalling pathway and tumorigenesis. Exp. Ther. Med. 19, 1997–2007. doi: 10.3892/etm.2020.8454, PMID: 32104259 PMC7027163

[ref10] Hou L. Zhao M. Huang C. He Q. Zhang L. Zhang J. (2021). Alternative oxidase gene induced by nitric oxide is involved in the regulation of ROS and enhances the resistance of *Pleurotus ostreatus* to heat stress. Microb. Cell Factories 20:137. doi: 10.1186/s12934-021-01626-y, PMID: 34281563 PMC8287771

[ref11] Hu Y. Stenlid J. Elfstrand M. Olson A. (2013). Evolution of RNA interference proteins dicer and argonaute in *Basidiomycota*. Mycologia 105, 1489–1498. doi: 10.3852/13-171, PMID: 23928424

[ref12] Jia X. Bi Y. Li J. Xie Q. Yang H. Liu W. (2015). Cellular microRNA miR-26a suppresses replication of porcine reproductive and respiratory syndrome virus by activating innate antiviral immunity. Sci. Rep. 5:10651. doi: 10.1038/srep10651, PMID: 26013676 PMC4445041

[ref13] Jones-Rhoades M. W. Bartel D. P. Bartel B. (2006). MicroRNAs and their regulatory roles in plants. Annu. Rev. Plant Biol. 57, 19–53. doi: 10.1146/annurev.arplant.57.032905.105218 16669754

[ref14] Kanehisa M. Goto S. Kawashima S. Okuno Y. Hattori M. (2004). The KEGG resource for deciphering the genome. Nucleic Acids Res. 32, 277D–2280D. doi: 10.1093/nar/gkh063, PMID: 14681412 PMC308797

[ref15] Kang K. Zhong J. Jiang L. Liu G. Gou C. Y. Wu Q.. (2013). Identification of microRNA-like RNAs in the filamentous fungus *Trichoderma reesei* by solexa sequencing. PLoS One 8:e76288. doi: 10.1371/journal.pone.0076288, PMID: 24098464 PMC3788729

[ref16] Kartha R. V. Subramanian S. (2014). Competing endogenous RNAs (ceRNAs): new entrants to the intricacies of gene regulation. Front. Genet. 5:8. doi: 10.3389/fgene.2014.00008, PMID: 24523727 PMC3906566

[ref17] Kim H. J. Chen C. B. Kabbage M. Dickman M. B. (2011). Identification and characterization of *Sclerotinia Sclerotiorum* NADPH oxidases. Appl. Environ. Microbiol. 77, 7721–7729. doi: 10.1128/Aem.05472-11, PMID: 21890677 PMC3209176

[ref18] Kiriga W. J. Chunyi Z. Jun L. Huan W. (2020). Plant non-coding RNAs: origin, biogenesis, mode of action and their roles in abiotic stress. Int. J. Mol. Sci. 21:8401. doi: 10.3390/ijms21218401, PMID: 33182372 PMC7664903

[ref19] Koonin E. V. Fedorova N. D. Jackson J. D. Jacobs A. R. Krylov D. M. Makarova K. S.. (2004). A comprehensive evolutionary classification of proteins encoded in complete eukaryotic genomes. Genome Biol. 5:R7. doi: 10.1186/gb-2004-5-2-r7, PMID: 14759257 PMC395751

[ref20] Kumar S. Stecher G. Tamura K. (2016). MEGA7: molecular evolutionary genetics analysis version 7.0 for bigger datasets. Mol. Biol. Evol. 33, 1870–1874. doi: 10.1093/molbev/msw054, PMID: 27004904 PMC8210823

[ref21] Langmead B. Trapnell C. Pop M. Salzberg S. L. (2009). Ultrafast and memory-efficient alignment of short DNA sequences to the human genome. Genome Biol. 10:R25. doi: 10.1186/gb-2009-10-3-r25, PMID: 19261174 PMC2690996

[ref22] Lara-Rojas F. Sanchez O. Kawasaki L. Aguirre J. (2011). *Aspergillus nidulans* transcription factor AtfA interacts with the MAPK SakA to regulate general stress responses, development and spore functions. Mol. Microbiol. 80, 436–454. doi: 10.1111/j.1365-2958.2011.07581.x, PMID: 21320182 PMC3108070

[ref23] Lee H. C. Li L. Gu W. Xue Z. Crosthwaite S. K. Pertsemlidis A.. (2010). Diverse pathways generate microRNA-like RNAs and dicer-independent small interfering RNAs in fungi. Mol. Cell 38, 803–814. doi: 10.1016/j.molcel.2010.04.005, PMID: 20417140 PMC2902691

[ref24] Li B. Cheng X. S. Zhang T. Liu L. L. Nie Z. M. Sheng Q. (2016). The identification of microRNAs in *Ganoderma lingzhi sporocarp*. Mycoscience 57, 271–278. doi: 10.1016/j.myc.2016.03.004

[ref25] Mao X. Cai T. Olyarchuk J. G. Wei L. (2005). Automated genome annotation and pathway identification using the KEGG Orthology (KO) as a controlled vocabulary. Bioinformatics 21, 3787–3793. doi: 10.1093/bioinformatics/bti430, PMID: 15817693

[ref26] Meng L. Lyu X. M. Shi L. L. Wang Q. J. Wang L. Zhu M. J.. (2021). The transcription factor FvHmg1 negatively regulates fruiting body development in winter mushroom *Flammulina velutipes*. Gene 785:145618. doi: 10.1016/j.gene.2021.145618, PMID: 33775849

[ref27] Moran Y. Agron M. Praher D. Technau U. (2017). The evolutionary origin of plant and animal microRNAs. Nat. Ecol. Evol. 1:27. doi: 10.1038/s41559-016-0027, PMID: 28529980 PMC5435108

[ref28] Mu D. S. Li C. Y. Shi L. Zhang X. C. Ren A. Zhao M. W. (2015). Bioinformatic identification of potential MicroRNAs and their targets in the Lingzhi or Reishi medicinal mushroom *Ganoderma lucidum* (higher basidiomycetes). Int. J. Med. Mushrooms 17, 783–797. doi: 10.1615/IntJMedMushrooms.v17.i8.80 26559864

[ref29] Qiu Z. Wu X. Gao W. Zhang J. Huang C. (2018). High temperature induced disruption of the cell wall integrity and structure in *Pleurotus ostreatus* mycelia. Appl. Microbiol. Biotechnol. 102, 6627–6636. doi: 10.1007/s00253-018-9090-6, PMID: 29846777

[ref30] Rehmsmeier M. Steffen P. Hochsmann M. Giegerich R. (2004). Fast and effective prediction of microRNA/target duplexes. RNA 10, 1507–1517. doi: 10.1261/rna.5248604, PMID: 15383676 PMC1370637

[ref31] Rocha C. R. C. Schroppel K. Harcus D. Marcil A. Dignard D. Taylor B. N.. (2001). Signaling through adenylyl cyclase is essential for hyphal growth and virulence in the pathogenic fungus *Candida albicans*. Mol. Biol. Cell 12, 3631–3643. doi: 10.1091/mbc.12.11.3631, PMID: 11694594 PMC60281

[ref32] Safa A. Abak A. Shoorei H. Taheri M. Ghafouri-Fard S. (2020). MicroRNAs as regulators of ERK/MAPK pathway: a comprehensive review. Biomed. Pharmacother. 132:110853. doi: 10.1016/j.biopha.2020.110853, PMID: 33068932

[ref33] Shao Y. Tang J. Chen S. Wu Y. Wang K. Ma B.. (2019). milR4 and milR16 mediated fruiting body development in the medicinal fungus *Cordyceps militaris*. Front. Microbiol. 10:83. doi: 10.3389/fmicb.2019.00083, PMID: 30761116 PMC6362416

[ref34] Shao J. Wang L. Liu Y. Qi Q. Wang B. Lu S.. (2020). Identification of milRNAs and their target genes in *Ganoderma lucidum* by high-throughput sequencing and degradome analysis. Fungal Genet. Biol. 136:103313. doi: 10.1016/j.biopha.2020.110853, PMID: 31751775

[ref35] Tatusov R. L. Galperin M. Y. Natale D. A. Koonin E. V. (2000). The COG database: a tool for genome-scale analysis of protein functions and evolution. Nucleic Acids Res. 28, 33–36. doi: 10.1093/nar/28.1.33, PMID: 10592175 PMC102395

[ref36] Teotia S. Singh D. Tang X. Q. Tang G. L. (2016). Essential RNA-based technologies and their applications in plant functional genomics. Trends Biotechnol. 34, 106–123. doi: 10.1016/j.tibtech.2015.12.001, PMID: 26774589

[ref37] Thompson J. D. Higgins D. G. Gibson T. J. (1994). Improving the sensitivity of progressive multiple sequence alignment through sequence weighting, position-specific gap penalties and weight matrix choice. Nucleic Acids Res. 22, 4673–4680. doi: 10.1093/nar/22.22.4673, PMID: 7984417 PMC308517

[ref38] Wang G. Li M. Zhang C. Zhan N. Cheng H. Gao Y.. (2021). Identification of microRNA-like RNAs in *Cordyceps guangdongensis* and their expression profile under differential developmental stages. Fungal Genet. Biol. 147:103505. doi: 10.1016/j.fgb.2020.103505, PMID: 33347973

[ref39] Wu T. J. Hu C. C. Xie B. G. Wei S. L. Zhang L. Zhu Z. X.. (2020). A putative transcription factor LFC1 negatively regulates development and yield of winter mushroom. Appl. Microbiol. Biotechnol. 104, 5827–5844. doi: 10.1007/s00253-020-10642-8, PMID: 32356196

[ref40] Xiao X. Tang Z. Li X. Hong Y. Li B. Xiao W.. (2017). Overexpressing OsMAPK12-1 inhibits plant growth and enhances resistance to bacterial disease in rice. Funct. Plant Biol. 44, 694–704. doi: 10.1071/Fp16397, PMID: 32480599

[ref41] Xiao S. Yang M. Yang H. Chang R. Fang F. Yang L. (2018). miR-330-5p targets SPRY2 to promote hepatocellular carcinoma progression via MAPK/ERK signaling. Oncogene 7:90. doi: 10.1038/s41389-018-0097-8, PMID: 30464168 PMC6249243

[ref42] Xu M. Li J. Wang X. Meng S. Shen J. Wang S.. (2018). MiR-22 suppresses epithelial-mesenchymal transition in bladder cancer by inhibiting snail and MAPK1/slug/vimentin feedback loop. Cell Death Dis. 9:209. doi: 10.1038/s41419-017-0206-1, PMID: 29434190 PMC5833802

[ref43] Xu D. Y. Zhou Q. X. Yan B. Y. Ma A. M. (2021). Identification and physiological function of one microRNA (Po-MilR-1) in oyster mushroom *Pleurotus ostreatus*. Mycoscience 62, 182–188. doi: 10.47371/mycosci.2021.01.004 37091326 PMC9157778

[ref44] Yang Q. Li L. Xue Z. Ye Q. Zhang L. Li S.. (2013). Transcription of the major neurospora crassa microRNA-like small RNAs relies on RNA polymerase III. PLoS Genet. 9:e1003227. doi: 10.1371/journal.pgen.1003227, PMID: 23349642 PMC3547838

[ref45] Yi J. Lee Y. S. Lee D. Y. Cho M. H. Jeon J. S. An G. (2016). OsMPK6 plays a critical role in cell differentiation during early embryogenesis in *Oryza sativa*. J. Exp. Bot. 67, 2425–2437. doi: 10.1093/jxb/erw052, PMID: 26912801 PMC4809295

[ref46] Zdanowicz A. Thermann R. Kowalska J. Jemielity J. Duncan K. Preiss T.. (2009). Drosophila miR2 primarily targets the m(7)GpppN cap structure for translational repression. Mol. Cell 35, 881–888. doi: 10.1016/j.molcel.2009.09.009, PMID: 19782035

[ref47] Zeng W. Wang J. Wang Y. Lin J. Fu Y. Xie J.. (2018). Dicer-like proteins regulate sexual development via the biogenesis of perithecium-specific micrornas in a plant pathogenic fungus *Fusarium graminearum*. Front. Microbiol. 9:818. doi: 10.3389/fmicb.2018.00818, PMID: 29755439 PMC5932338

[ref48] Zhang J. J. Hao H. B. Wu X. L. Wang Q. Chen M. J. Feng Z. Y.. (2020). The functions of glutathione peroxidase in ROS homeostasis and fruiting body development in *Hypsizygus marmoreus*. Appl. Microbiol. Biotechnol. 104, 10555–10570. doi: 10.1007/s00253-020-10981-6, PMID: 33175244

[ref49] Zhang J. Liu H. Lin H. Li S. C. Tao H. H. Zhang L.. (2017). Sp1 is a competitive endogenous RNA of Klf4 during odontoblast differentiation. Int. J. Biochem. Cell B. 85, 159–165. doi: 10.1016/j.biocel.2017.02.008, PMID: 28238937

[ref50] Zhang H. Sun P. Wang Y. L. Yu X. F. Tong J. J. (2020). MiR-214 promotes proliferation and inhibits apoptosis of oral cancer cells through MAPK/ERK signaling pathway. Eur. Rev. Med. Pharmacol. Sci. 24, 3710–3716. doi: 10.26355/eurrev_202004_20834, PMID: 32329847

[ref51] Zhou J. H. Fu Y. P. Xie J. T. Li B. Jiang D. H. Li G. Q.. (2012). Identification of microRNA-like RNAs in a plant pathogenic fungus *Sclerotinia sclerotiorum* by high-throughput sequencing. Mol. Gen. Genomics. 287, 275–282. doi: 10.1007/s00438-012-0678-8, PMID: 22314800

[ref52] Zhou Q. Wang Z. Zhang J. Meng H. Huang B. (2012). Genome-wide identification and profiling of microRNA-like RNAs from *Metarhizium anisopliae* during development. Fungal Biol. 116, 1156–1162. doi: 10.1016/j.funbio.2012.09.001, PMID: 23153806

