# Corrigendum: milR20 negatively regulates the development of fruit bodies in *Pleurotus cornucopiae*

**DOI:** 10.3389/fmicb.2023.1236756

**Published:** 2023-07-12

**Authors:** Yuhui Qi, Chenyang Huang, Mengran Zhao, Xiangli Wu, Guangyu Li, Yingjie Zhang, Lijiao Zhang

**Affiliations:** ^1^Institute of Agricultural Resources and Regional Planning, Chinese Academy of Agricultural Sciences, Beijing, China; ^2^Key Laboratory of Microbial Resources, Ministry of Agriculture and Rural Affairs, Beijing, China; ^3^State Key Laboratory of Efficient Utilization of Arid and Semi-arid Arable Land in Northern China, Beijing, China; ^4^College of Life Sciences, Shanxi Normal University, Taiyuan, China

**Keywords:** milR20, fruit body development, *Pleurotus cornucopiae*, comparative transcriptome, MAPK signaling pathway

In the published article, there was an error in Figure 5 as published. The gene in Figure 5E was displayed as “g3400”. The correct statement is “g10683”. The gene in Figure 5F was displayed as “g3400”. The correct statement is “g7031”. The corrected Figure 5 and its caption appear below.

**Figure 5:**
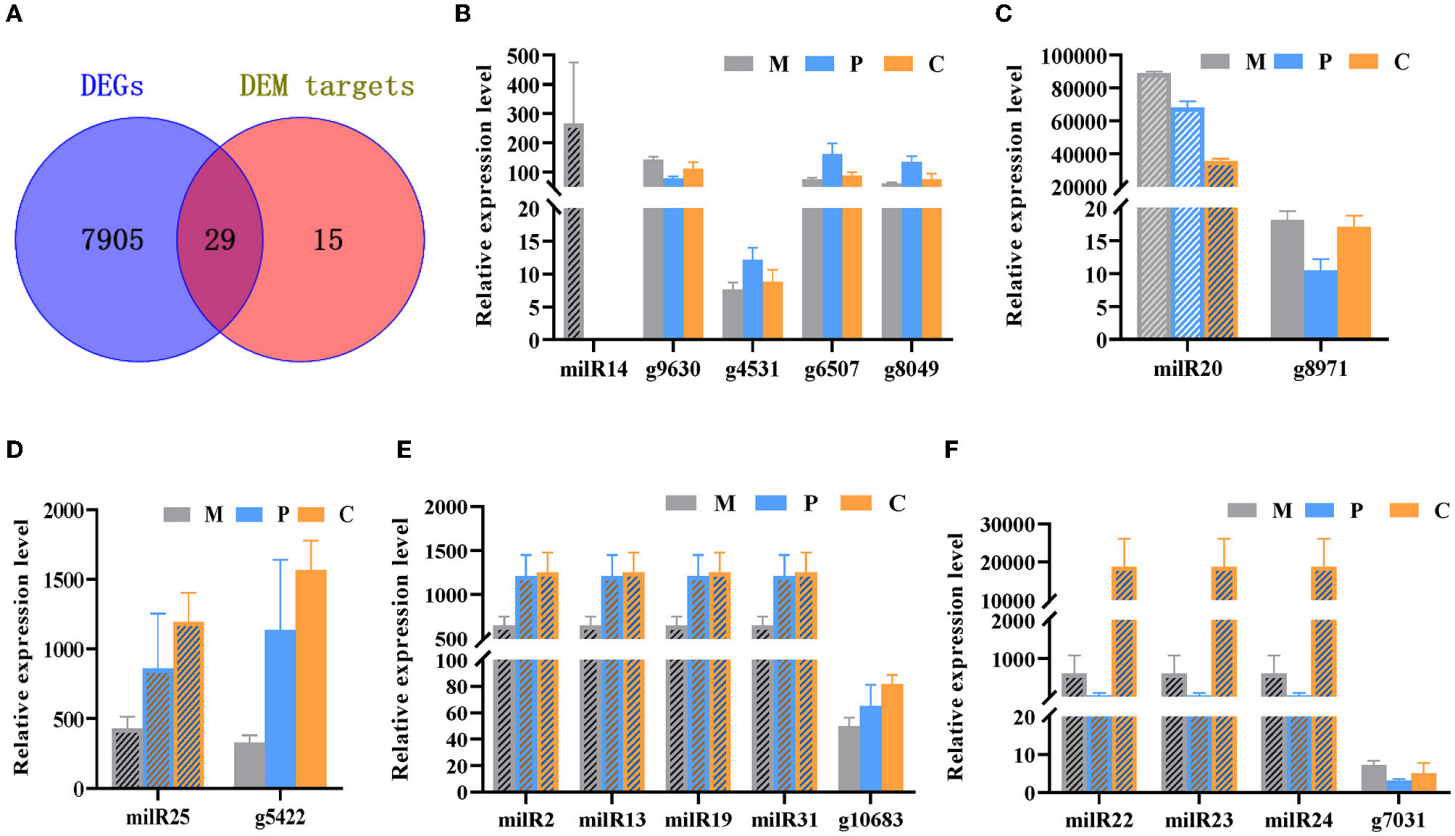
Integrated analyses of mRNA and milRNA data. **(A)** Venn diagram depicting the DEGs in the different stages of development and DEM targets. **(B–F)** Analysis of the expression levels of the DEMs and their targets DEGs.

In the published article, there was an error in the Funding statement. The names of the first two funding bodies were incorrectly presented as “Fundamental Research Funds for China Agriculture Research System” and “Central Nonprofit Scientific Institution”. The correct Funding statement appears below.

