# Supplementary material for: milR20 negatively regulates the development of fruit bodies in Pleurotus cornucopiae
Source: Front Microbiol. 2023 May 4;14:1177820. doi: 10.3389/fmicb.2023.1177820 (PMC10192896; doi:10.3389/fmicb.2023.1177820)
Supplement: Supplementary file 2 [file Data_Sheet_1.DOCX]

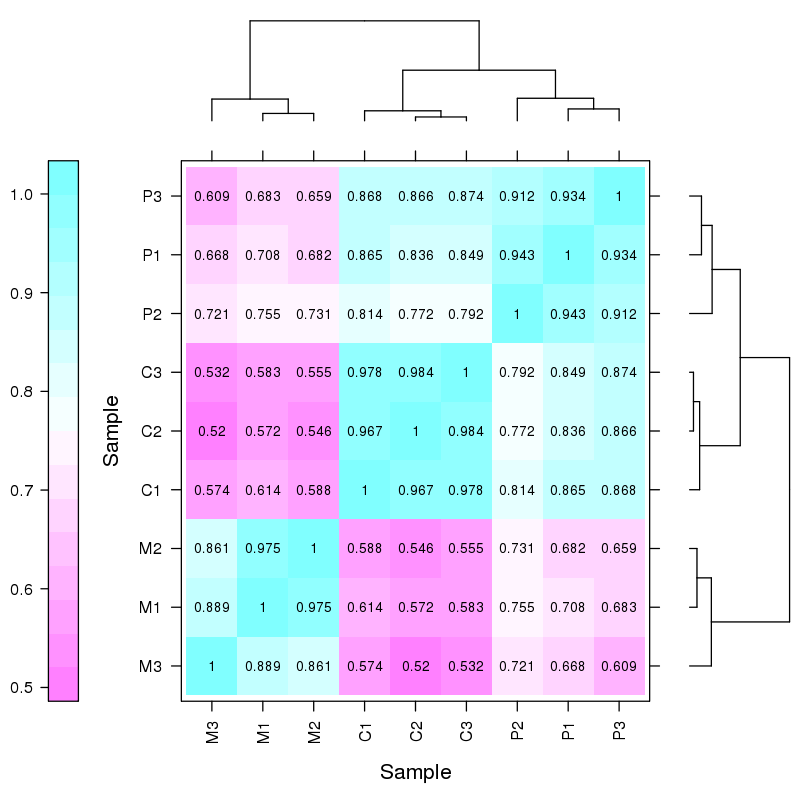


Figure S1 The pearson correlation coefficient between mRNA sequencing samples. M: mycelia; P: primordia; C: cap of fruiting bodies.


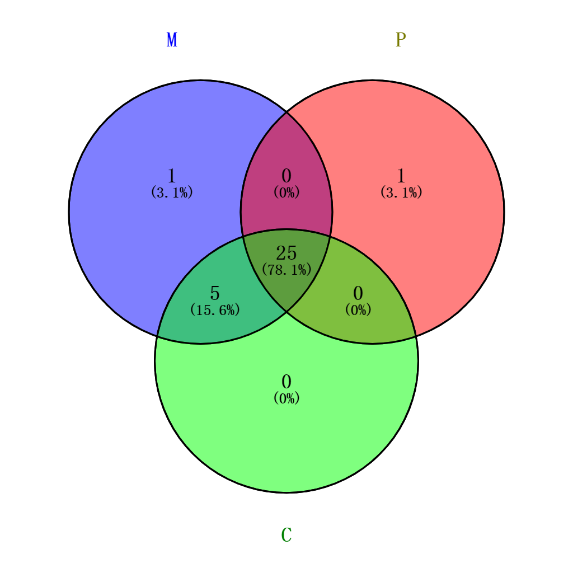


Figure S2 The venn diagrams of milRNAs identified in the three developmental stages of. M: mycelia; P: primordia; C: cap of fruiting bodies.


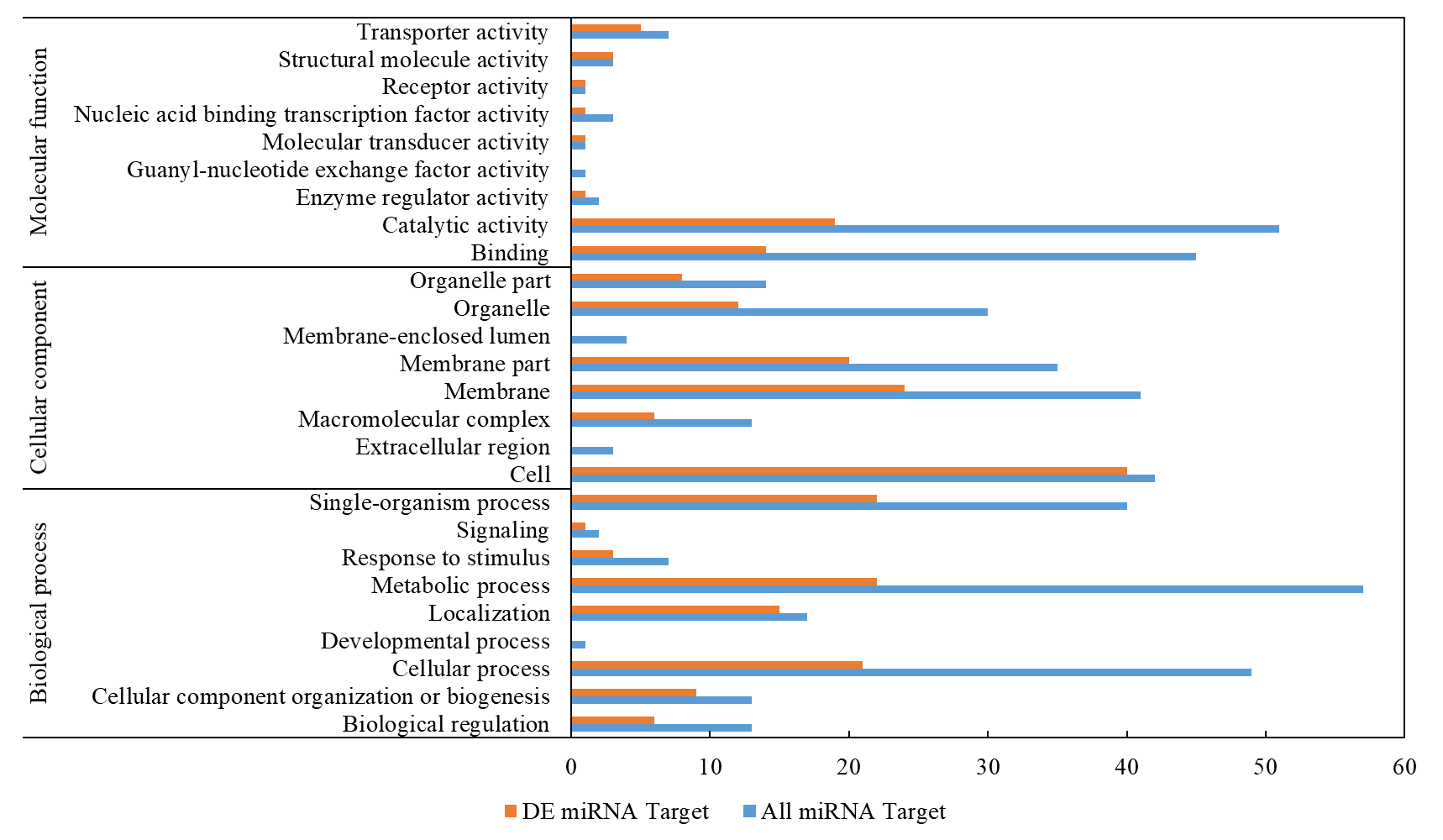


Figure S3 GO enrichment analysis of target genes of all DEMs

Figure S4 qRT-PCR verification of the expression patterns of the g8971 and milR20. (A) Relative expression level of g8971 in qRT-PCR experiment and mRNA-Seq. (B) Relative expression level of milR20 in qRT-PCR experiment and milRNA-Seq. The qRT-PCR data are presented as mean ± standard error of mean of three independent experiments.


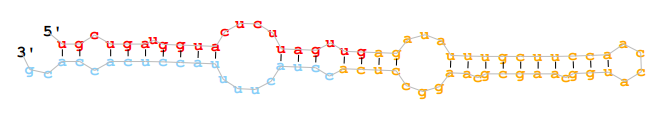


Figure S5 Hairpin structure of pre-milR20

Sequence in red is mature milRNAs, whereas sequence in purple is the milRNAs*, and the sequence in yellow is the stem-loop region.

Figure S6 The effect of milR20 on the primordium formation time.
